# Power Dynamics Among Health Professionals in Nigeria: A Case Study of the Global Fund Policy Process

**DOI:** 10.34172/ijhpm.2022.6097

**Published:** 2022-04-19

**Authors:** Samuel Lassa, Muhammed Saddiq, Jenny Owen, Christopher Burton, Julie Balen

**Affiliations:** ^1^School of Health and Health Related Research, University of Sheffield, Sheffield, UK.; ^2^Academic Unit of Primary Medical Care, University of Sheffield, Sheffield, UK.; ^3^The Department of Community Medicine, University of Jos, Jos, Nigeria.

**Keywords:** Global Health, Power Dynamics, Nigeria, Sociology of Professions

## Abstract

**Background:** Health workers are central to health policy-making. Given health systems’ complex, dynamic and political nature, various forms of ‘hidden power’ are at play as health workers navigate health systems. This study aims to explore the dynamics of power and its sources, and how this shapes policy-making and implementation within the Nigerian health systems context.

**Methods:** The case study was the Global Fund grant in Nigeria, and results are based on an in-depth qualitative study involving 34 semi-structured key informant interviews (KIIs), board-meeting observations, and documentary analysis conducted in 2014 and 2016. Participants held mid to senior-level positions (eg, Director, Programme Manager) within organisations involved with Global Fund activities, particularly proposal development and implementation. Data were analysed using thematic analysis in order to gain insight into the power dynamics of health professionals in policy processes.

**Results:** Medical professionals maintained dominance and professional monopoly, thereby controlling policy spaces. The structural and productive power of the biomedical discourse in policy-making encourages global actors and the local government’s preference for rapid biomedical models that focus on medications, test kits, and the supply of health services, while neglecting aspects that would help us better understand the poor uptake of these services by those in need. The voices of the repressed groups (eg, non-clinical experts, patients and community based organisations) that better understand barriers to uptake of services are relegated.

**Conclusion:** Professional monopoly theories help illustrate how medical professionals occupy and maintain an elite position in the health system of Nigeria. Structural and agential factors specific to the contexts are key in maintaining this professional monopoly while limiting the opportunities for other health occupations’ rise up the social status ladder.

## Background

**Key Messages**

** Implications for policy makers**

- This paper highlights how the theories from the sociology of professions help explain the medical professional dominance of the health policy space in Nigeria.
- This study contributes to empirical literature from low- and middle-income countries (LMICs) on the multiple sources of biomedical dominance that contribute to medical professionals’ influence in health policy and implementation.
- Findings show that existing professional hierarchies can capitalise on multi-sectorial platforms, such as the Global Fund’s Country Coordinating Mechanism (CCM) to enhance their influence in the health system. Policy-makers and global health partners need to be aware of their roles in perpetuating power imbalance and the need to promote a more multi-sectoral approach for health planning to mitigate decision-making monopoly.
- Health policy-makers face several constraints in achieving holistic system-wide health strategies in a health system dominated by the biomedical discourse. Policy spaces have to be opened up to allow active participation of repressed interests that represent both the patient population and other non-clinical professions. This can be made possible through the sensitisation of the non-health sector and the removal of structural barriers that perpetuate professional monopolies and create obstacles to broader multi-disciplinary and systems thinking approaches.

** Implications for the public**

With most global health actors focused on improving access and decentralisation of care in low- and middle-income countries (LMICs) settings, competencies that are unique to medical professionals that make them effective in carrying out their roles can be introduced into the curriculum of other health professionals. Evidence from health challenges, such as HIV/AIDS, malaria, tuberculosis (TB) and the current coronavirus disease 2019 (COVID-19) pandemic have shown why we need to recognise the optimisation of services of all health professionals, and seek ways to professionalise and engage them in future health policy discourse. This will create a health workforce that can effectively and efficiently complement medical professionals in health policy formulation and implementation.

In health policy literature, policy failure^1^ has been attributed to, among other factors, a lack of multidisciplinarity and ‘systems thinking,’ including at the level of policy-making, policy implementation, policy outcomes and policy research.^2-4^ Ooms noted that the biggest challenge to multi-disciplinary problem-solving in global health is the strong preference towards a biomedical discourse in the conceptualisation of health by various actors. Indeed, historical drivers of the conceptualisation of health have given rise to the contemporary dominance of the biomedical reductionist paradigm and discourse, with overwhelming imbalances that continue to suppress multi-disciplinary problem solving and policy success.^5^ In the emerging field of health policy and systems research, a multi-disciplinary approach to health policy-making in low- and middle-income countries (LMICs) has been gaining attention, particularly in relation to understanding power and power dynamics among different actors^[1]^.^6^

 Complicated power dynamics do exist among different health actors, and while in some cases they are apparent,^5^ in others they may remain tacit or even hidden – including to those who use them and benefit from them.^7^ Research into power, policy and health systems in LMICs has revealed that ‘in country’ medical professionals are key to health policy processes^2^ and that they influence policy feasibility and its overall success or failure.^8-10^ However, when concentrated in the hands of a few actors, such as medical doctors, such power to influence policy processes can deepen preferences for the use of the biomedical paradigm in shaping the health agenda, thereby creating further obstacles to broader multi-disciplinary and systems thinking approaches.^11,12^ Empirical studies on such issues remain limited, with this area of research only recently gaining attention among many global health scholars.^12^ There is an urgent need to better understand various forms of power, including the hidden power of discourses, and to explore and uncover how power is distributed and exercised at the country level. This will help mitigate its more disruptive effects, such as policy failure of costly Global Health Initiatives (GHIs).^7,13-15^

 We draw similarities between some of the theoretical explanations of the forms of power and the sociology of professions to provide insight into the power dynamics that exist among health professionals. Shiffman, for example, states that *‘epistemic and normative (power) invoke both structural and productive power’*^13^ (p. 297). and that this structural power is seen *‘in the existence of a cadre of individuals’*^13^ (p. 297). This can be likened to occupational hierarchies in the health sector, as described in the sociology of professions.^16^ Productive power is said to *‘create concepts for thinking about health priority-setting,’*^13^ (p. 297) and can be compared to the historical monopoly that medical professionals had (and continue to have) over knowledge creation in the health sector.^17^ By drawing on ideas from the sociology of professions, global health researchers can thus begin to explore the influence of biomedical power in national health policy-making in LMICs. Moreover, tracing the influence of (powerful) GHIs, such as the Global Fund for example, is also critical, including whether and how they help maintain these organised biomedical structures and in turn perpetuate the dominance of the medical profession. Dalglish et al,^8^ captured this empirically when showing how medical power can be used to steer health system priorities in India and Niger, leading to the medicalisation of public health issues.

 This study uses the Global Fund’s Country Coordinating Mechanism (CCM) as a case study, since it is a notable platform from which to explore the role of multi-disciplinary problem-solving in global health policy processes. The CCM constitutes an open and multi-stakeholder platform, with diverse professional groups drawn from across the health system.^18^ Moreover, CCMs are central to the Global Fund’s commitment to local ownership and participatory decision-making, and they include representatives from both the public and private sectors, including governments, multilateral or bilateral agencies, non-governmental organizations, academic institutions, private businesses and people living with the diseases that the policies target.^19^ This country-level multi-stakeholder partnership develops and submits grant proposals to the Global Fund based on priority needs at the national level. Upon grant approval, they oversee progress during implementation.^19^ Here, we explore health professional interactions within the Global Fund’s CCM in Nigeria, with the aim of explaining dynamics of power and its sources, and how this shapes policy-making and implementation within the Nigerian health system context.

###  The Case

 The single largest source of funding for health in Nigeria is out-of-pocket payment, accounting for 77% of overall financing, followed by the government with 14%, and development partners, such as the Global Fund, President Emergency Plan for AIDS Relief and Department for International Development with a total of 8%.^20^ While the proportion of health expenditure by development partners is relatively small, the concentration of these funds on three disease entities, namely HIV/AIDS, tuberculosis (TB) and malaria, means that GHI programmes and policies within the country play a major policy role in the three biggest communicable causes of morbidity in Nigeria.^21^ The open platform and multi-stakeholder policy-making process of the Global Fund’s CCM^22,23^ makes it the most favourable case study in Nigeria to explore the interactions of local health system actors with such GHIs. Data were collected between January and June 2016 in Abuja, with the unit of the study identified as the CCM.

 In 2002, the Nigerian CCM submitted its Round 1 Global Fund proposal with a focus on the expansion of Prevention of Mother to Child Transmission of HIV and the creation of Prevention of Mother to Child Transmission of HIV centres of excellence around the country to control the HIV/AIDS epidemic.^24^ Since then Nigeria has successfully applied for several Global Fund grants worth over US$1 billion focussing on tackling HIV/AIDS, TB and malaria, and Global Fund’s particular interest in Nigeria is driven by the fact that in 2016 Nigeria had the second-highest number of people living with HIV/AIDS, which made tackling HIV/AIDS in Nigeria one of the Global Fund’s top priorities. Several vertical and diagonal strategies ranging from a focus on cross-cutting health system strengthening interventions, to scale up of antiretroviral (ARV) treatment have been implemented through the Global Fund grants, however an Office of the Inspector General (OIG) report in 2016 showed that the grant was not fully effective in all areas of programme implementation.^25^

## Methods

 A critical realist epistemic standpoint was used in exploring the dynamics of power, and its sources, and how this shapes policy-making and implementation within the Nigerian health system context.^26^ Critical realism is another perspective in the health policy and systems research knowledge paradigm spectrum.^26^ Critical realists ‘seek to explain change by referring to the actors who change a situation under influence of particular external events (such as an intervention) and under specific conditions.’^27^ The case study design allowed for an in-depth investigation of processes and interactions in one specific policy context.^28^ This case study was exploratory in nature and Yin stated that ‘how’ and ‘why’ questions are explanatory in nature and are asked about an existing set of events, over which the researcher has little or no power.^28^ The study used three complementary data sources: key informant interviews (KIIs), non-participatory observation, and documentary data. However, the main source of data was the KII.

###  Interviews

 The purposive sampling used was informed by the theoretical framework, which was a product of the literature review, the research questions and the documents gathered prior to fieldwork. This information, with the CCM’s list of stakeholders, was used to identify the first set of participants from different professional backgrounds through a maximum variation sampling strategy (using professional background), with the aim of achieving variability within the primary data. During interviews, participants were asked if they knew past CCM members or other people who had insight about the Global Fund grant and the topic of research.

 Semi-structured KIIs were carried out with directors, programme managers and patient group leaders holding positions within organisations involved in activities of the Global Fund proposal process, particularly at the proposal development stages and implementation. The process led to the inclusion and addition of past members not included in the initial list, such as any past Global Fund portfolio members for Nigeria. This helped to limit the selection bias and recall bias, because past CCM members and partners could have potentially been left out in the initial sampling list. Written informed consent was obtained, and all interviews were conducted face to face. Results were based on 34 interviews consisting of the initial set of 23 participants that were drawn up by the research team and 11 others who were identified during the course of the first set of interviews, with each interview pointing the researcher to relevant sources of data until data saturation. The interview guide asked open-ended questions about participation during proposal writing stages of current and previous Global Fund grant applications. The data were transcribed verbatim by an independent person, and all members of the research team familiarised themselves with the data by reviewing the audio recordings and transcript.

###  Non-participatory Observations

 The principal researcher carried out two non-participatory observations: the first was a large CCM meeting involving CCM members and non-voting stakeholders, which was done concurrently with the interviews, while the second observation was a smaller technical proposal writing meeting carried out at the end of data collection. The observation process focused on the verbal and non-verbal communications between the participants at the meetings as well as the content of the meeting. The observations had the overall aim of identifying gaps or inconsistencies in interviewee accounts.

###  Documentary Data

 Three members of the research team carried out documentary analysis of 63 documents from 2014 to 2018 to corroborate findings from other sources of data, understand the style, codes, and language used during meetings, in addition to identifying topics that needed to be probed while interviewing participants. The types of documents needed for this part of the study were identified through interactions with staff of the CCM and other key informants interviewed during the course of the study. Relevant material evidence collected from actors in the policy process included previous proposals applied for by Nigeria, the old and new guidelines for the grant writing process, minutes of meetings and evaluation reports. Access to these documents was obtained through the CCM and the Global Fund website.

###  Analysis

 The six steps of thematic analysis according to Braun and Clark was used to analyse the interview data through an inductive iterative process of identifying themes.^29^ The interview data as the primary source of data were independently coded by the principal researcher and two members of the research team. The final themes generated from the interview data guided how the document data and observational data were coded and analysed. Convergence, inconsistencies and contradictions were explored in this stage of analysis. For example, the theme ‘Wasted ARVs’ generated from the interview data were explored in the document data from the 2016 OIG report to confirm the claims made by participants. Similarly, the theme ‘Power in collective numbers’ was identified in documentary data of CCM minutes and observational data. This method of triangulation used the data sources, documentary and observational, to create a comprehensive and rich account of the interview data on the power dynamics of health professionals in Nigeria. In the final stage of interpretation, the concepts highlighted below were used to explain the sources of power. The works of Barnett and Duvall on the forms of power,^30^ which are institutional, productive and structural power, informed the concepts used as summarised in Table 1. However, in the analysis of this paper, we have only identified instances of productive and structural power (see discussion section). Table 1 also highlights intersections of these sources of power with Freidson’s sociology of professions.^16^ Freidson’s theories on professional hierarchy in favour of doctors and their privileged position as custodians of biomedical knowledge aligns respectively with Barnett and Duvall’s structural and productive power. The results section uses the Gill Walt and Lucy Gilson’s ‘Triangle Framework’ for health policy analysis^31^ to represent the views of participants, observations and documentary evidence. This will form the basis of the discussion section.

**Table 1:** Conceptual Framework

**Forms of Power, According to Barnett and Duvall**	**Mechanisms Used by Professionals Linked to Concepts FromFreidson’s Sociology of Professions** ^16,32^
Structural power: *“Structures allocate differential capacities, and typically differential advantages, to different positions”*^30^ (p. 53).	Professional monopoly creates an occupational hierarchy, which differentiates privileges, limiting it to certain roles and strategic positions in the health sector.
Productive power: *“Concerns discourse, the social processes and the systems of knowledge through which meaning is produced, fixed, lived, experienced, and transformed”*^30^ (p. 55).	Medical professionals are regarded as biomedical experts, which hence positions them as dominant actors in the framing of health priorities during policy processes.

## Results

###  Description of Participants

 Participants (n = 34) all held positions within organisations involved with Global Fund activities, particularly at the proposal development and implementation stages. The majority of participants were programme managers, whose job descriptions ranged from carrying out Global Fund activities for their various organisations during implementation, to being hired as consultants in the Global Fund proposal writing process. Others were directors who were the highest decision-making cadres in their organisations, overseeing the entire organisation’s programme management. Consultants who were brought into the Global Fund programme by the CCM for their expertise in areas lacking by the members and participants were also interviewed. Half of the participants were medical doctors (n = 17) with the remaining 17 participants from other professions (Table 2). The median number of years working with the Global Fund grant among all participants was 4.5 years. The variation in professional background did not affect views about which professional groups were more dominant (ie, doctors) or whether the dominance of doctors in the policy process was justifiable. However, there were variations of views regarding the impact of this on policy, which is based on professional background.

**Table 2:** The Background Characteristics of the Study Participants

**Characteristic**	**Number (N = 34)**
Gender	
Male	22
Female	12
Professional background	
Medical professional	17
Finance expert	2
Health economist	1
Public health expert	4
Management expert	1
Monitoring and evaluation expert	3
Pharmacist	2
Social scientist	4
Position	
Programme manager	20
Consultant	2
Director	1
Deputy director	9
Member country proposal team	1
CCM secretariat	1
Sector	
Private	22
Public	12
Organisation	
INGO	12
Local non-governmental organisation	11
CCM Secretariat	1
Community-based Organisation	1
Patient group member	1
Government agency	7
Global Fund	1
Work experience (median years)	4.5 years
Range	1 year to 13 years

Abbreviations: CCM, Country Coordinating Mechanism; INGO, international non-governmental organisation.

Table 3 shows the Braun and Clark’s method of thematic analysis used in this study, which is a type of reflexive thematic analysis. Code building captures one observation, while the theme summarises multiple similar observations. The thematic patterns draws “*together several of these ‘themes’ (codes) into richer, more complex themes that revealed multiple facets of a particular meaning or experience.”*^33^

**Table 3:** The Thematic Analysis

**Research Questions: Are There Influential Actors in The Global Fund Policy Process in Nigeria? How Do They Influence the Processes? Why Do They Have This Influence?**				
Interview questions	What was your role in Global Fund Grant Nigeria?	Describe the level of your involvement in the affairs of the Global Fund proposal process and implementation?	Were there any influential actors in these processes?	How do they influence the processes? Why do they have this influence?
Primary codes generated from dataset	Director Patient activist Health economist Financial expert Public health expert	Selection of Principal Recipients for the grant RMC meetings Proposal writing meetings CCM stakeholder’s meeting	Financial experts Medical doctors RMC members INGO staff	Medical doctors with clinical and managerial roles Medical doctors dominate in numbers Use of professional language
Themes	Level of involvement Work experience	Global Fund Technical Review Panel influence on policy content Pre-determined policy objectives and procedures	INGOs run by medical doctors Process driven by medical doctors	Societal status of medical doctors Collective behaviour of medical doctors
Thematic patterns (complex themes)	Professional background of participants	Structural institutions with biomedical preference Biomedical dominated proposal contents Wasted ARVs	Medical doctors’ dominance in the Nigerian health system	Positional power and Occupational hierarchy Use of biomedical language Power in collective numbers

Abbreviations: CCM, Country Coordinating Mechanism; RMC, Resource Mobilisation Committee; INGO, international non-governmental organisation; ARVs, antiretrovirals.

###  Multiple Sources of Biomedical Dominance 

 There are seven main themes from Table 3 presented in the findings section. This study identified multiple sources of biomedical dominance in the policy process and these could be seen as contributory factors to the existing medical professional monopoly of health policy and implementation. In the context studied, we could identify three potential sources that linked biomedical dominance to professional monopoly; the number of doctors and the positions they hold, the interventions being considered and the guidelines that dominate the policy processes. Some of these sources are discussed in the themes below.

###  Positional Power and Occupational Hierarchy

 While participants noted that at the national level, more programme design and management skills are needed, medical doctors nonetheless dominated management and policy-making at all levels.

 “*…the medical professionals are leading most of the government health agencies and even the partners, which are also part of the decision making process. You basically have them leading the process in terms of decision making with regards to proposal writing priorities and all that. I mean that is a fact” *(Participant 28: Medical Doctor).

 Most participants, including doctors themselves, said that the Nigerian health system provides medical professionals with an advantage because the health system’s occupational hierarchy places medical professionals above all other occupations in both managerial and clinical roles.

 “*Well it is probably as a result of the way the national health system is managed in the sense that it is assumed that the doctors lead the team. So most times the doctor dictates or states how they want the programmes to run” *(Participant 3: Medical doctor).

 In addition, most participants’ accounts characterised medical professionals as ‘drivers’ of the proposal process and their views often supersede those of other non-medical professionals, patient groups and local community based organisations, who remained in more passive recipient roles. Even though there were various thematic areas in the proposals, the technical influence of this group of public health doctors as they were called, spreads across all thematic areas of the policy process.

 “*So mainly public health doctors looking at the trend, all the principal recipients for the Global Fund, most of the individuals at the helm of affairs and most of the individuals that are involved in decision making for the implementation of the grant, are mainly medical doctors with a public health background. Yes I can say that”* (Participant 17: Pharmacist).

###  Power in Collective Numbers

 The data suggests there was an awareness among participants that other non-biomedical viewpoints were accorded less weight during policy debates because medical professionals outnumbered other professionals during deliberative processes:

 “*I think it is because it is a game of numbers. Like when you have the lead people who are designing the overall strategy for the proposal, coming from one side [more] than the others, naturally this will happen” *(Participant 33: Health economist).

 It was apparent that professional monopoly by medical professionals in the Nigerian health system exists, and the Global Fund’s grant writing process was no exception. There was evidence to show that efforts were made to mitigate against medical professional monopoly in the Global Fund grant writing process. In addition, in subsequent CCM meetings for the Consolidated Global Fund grant, non-medical professionals were allocated more member seats in planning and technical committees, although not enough to attain a majority.

###  Medical Doctors’ Dominance in the Nigerian Health System

 Participants described how influence from doctors has, over time, spread into independent Public-Private Partnership programmes of the Global Fund’s implementing partners, such as Family Health Initiative (now known as FHI 360), Society for Family Health and Planned Parenthood Federation of Nigeria. Moreover, they noted that this in turn reproduces the medical power even in the health-related non-governmental organization sector in Nigeria. HIV/AIDS programmes were depicted as being prone to medical professional influence because HIV/AIDS has been categorised as a specialised medical field by most health sector institutions in Nigeria.

 “*Based on my experience so far with the Global Fund...people who have health backgrounds,… basically those who have medical backgrounds have more influence” *(Participant 19: Non-doctor monitoring and evaluation expert).

###  Use of Biomedical Language

 The content of interventions funded by Global Fund also favours biomedical approaches. The focus on clinical testing and ARV treatments with less emphasis on social interventions was one example of how biomedical content dominated proposals. This preference for biomedical evidence by the Global Fund’s Technical Review Panel^[2]^ (TRP) was cited as the main reason for using medical language during meeting proceedings. Here the source of medical doctors’ power is centred on the fact that they are considered more knowledgeable about the biomedical content needed by the TRP.

 Meetings were more often than not overshadowed by medical language and only those trained, experienced and confident in biomedical sciences could contribute to discussions, even though most times the topic of discussion had little to do with medical evidence or science.

 “*What I mean is that medical doctors use medical language, which does not lead to a meaningful discussion with other occupations during meetings. So when I say dominate, it is more about the type of language they use” *(Participant 30: Medical Doctor).

 “*All the experts gave their Epi-analysis showing the increasing trend of HIV/AIDS spread with an unmet need for ART at 57%. There was a feeling in the room that not all these reports were new information to the audience and it dwelled on scientific indices, and had little answers to behavioural patterns fuelling the increasing trend and poor access to health services that was leading to the low demand in the country. This prompted an influential personality to say ‘we need social science to push for better explanation of why (epi-analysis trends).’ This was then followed by a comment from a representative of a very influential organisation in support but this did little to change the direction of the meeting, which appeared to have a fixed agenda for more presentations from medical experts” *(Notes from observations of Global Fund meeting on the National and State epidemic impact analysis, March 6, 2014).

 This type of linguistic exclusion of non-medical stakeholders in meeting deliberations has the effect of centring discourse around biomedical disease prevention, thereby neglecting a more community oriented and broader multi-disciplinary approach relevant to the context. The synergistic effect of crowding out other opinions and the use of biomedical language in meetings, helps in shaping the health strategies in the grant proposals.

###  Biomedical Dominated Proposal Contents

 In sections of the proposal forms where recipient countries are requested to demonstrate supporting evidence, the Global Fund provides suggestions on the type of evidence to reference in those sections. For example, the Global Fund Information Note: *Strategic Investments for HIV Programs*, highlights specific intervention activities applying countries must capture in the proposals, linking them to various aims of the Global Fund.^34^ *‘It seems they have the answers to the questions they want you to answer’* (participant 32), such as the ‘test and treat’ strategy, which limits the engagement of local contextual knowledge when CCM members sit to develop proposals.

 “*Contextualising your country’s concept (proposal documents) actually does not come into play in Global Fund. Because...their rigid system is so rigid, everything is already spoon feeding (with suggested guidelines and strategies), that’s why I said, there is already a gutter (designed path) for you, so you must pass through” *(Participant 32: Non-doctor public health expert).

###  Structural Institutions With Biomedical Preference

 Documentary analysis indicated that high-level organisations recognised the importance of community mobilisation models of the social science disciplines, which can bring to light some of the contextual peculiarities in the country. Stakeholders in in-country proposals have highlighted this gap, where they have admitted that the country has no National Community Systems Strengthening (CSS) framework available. This is captured in the documentary extract below:

 “*More often than not, while government policies recognize the need for community systems to be mobilized for an all-inclusive process, the mechanism through CSO [Civil Society Organisation ] is given scant attention, this is responsible for non-availability of the National CSS framework” *(TB and HIV Concept Note Investing for impact against TB and HIV).^35^

 However, Global Fund processes that impact policy-making and implementation are structured by technical tasks or activities that maintain/protect the existing medical profession’s dominance. Some participants felt the overarching principles of the proposals were guided by the World Health Organization (WHO) and United Nations guidelines, and in situations where there had been conflicts or disagreements the WHO guideline was used as a reference point in making a final decision:

 “*The whole proposal writing process is governed by standardised principles or guidelines by WHO, UNAIDS (United Nations Programme on HIV and AIDS), so those serve as a reference point for finalisation of decisions” *(Participant 3: Medical doctor).

 In many instances, the WHO guidelines made it difficult to align the country’s contexts of CSS with the proposals. The WHO guidelines became a source of confusion rather than a useful tool. Participants were of the opinion that if the portfolio of the country is already set by the Global Fund with fixed budgets in such a way that ‘*a path is shaped for you to follow’ *(participant 22: Medical Doctor), then the process is already prescriptive and a lack of adherence to the rules leads to delays in TRP’s approval for the grant:

 “*Global Fund should not be prescriptive. They are too prescriptive. What they should do is to let us know how much you have, let us know the areas you want those monies to be spent, and those areas you want the monies to be spent should actually align with the country’s roadmap” *(Participant 22: Medical Doctor).

 In the critical stages of the proposal writing process before submission of the grant “*consultants are hired to help moderate, clean up the language and hand-over to CCM” *(Participant 21: Finance expert).Hired consultants (with medical backgrounds) from organisations, such as the WHO and UNAIDS influence the first stage, while members of the Resource Mobilisation Committee who control the final stage of the proposal draft were staff with similar medical backgrounds from influential organisations, such as WHO, UNAIDS, Clinton Health Access Initiative and United States Agency for International Development. Here, we identified another source of power, situated at the initial stages of policy formulation, whereby the processes are controlled by institutions who are led by medical professionals.

 The paradox in the policy process is the way in which during proposal writing the process removes integral elements of CSS captured in its planning stages because guidelines have to be followed, and consultants constantly try to align the proposal documents to meet international principles and standards. This can be called the process effect.

###  Wasted Antiretrovirals

 The result of this biomedically dominated agenda according to the participants has led to an overwhelming focus on clinical strategies during programme design because they were seen as being evidence-based, therefore making them hard to compete with or argue against. However, clinical judgement in most cases cannot predict operational challenges, and concerns were raised about the ongoing wastage of resources that results from such biomedically biased strategies.

 “*In fact, it is actually ridiculous sometimes when you are designing programmes, you want to include community components, and they say, no, no. Instead of doing community components, they say scale up ARVs, then you buy ARVs, at the end of the day, they will expire and then you go and pay people to go and destroy the expired drugs. Because there is no demand” *(Participant 21: Finance expert).

 Evidence of medical supply wastage due to low uptake of supplies in the community is also noted in an OIG 2016 report.

 “*As a result, the OIG noted 20 tons of expired HIV commodities at the central medical store, most of which were Global Fund purchased commodities and 15 tons at the state medical stores which have accumulated since 2005. The value of those commodities couldn’t be calculated due to the state these drugs were stored: Audit Report: Global Fund Grants to the Federal Republic of Nigeria”*^25^ (p. 13).

 While the vision behind Global Fund’s CCM is that of an open platform where different professional disciplines and lay members of the public have an equal say in policy-making and implementation, in Nigeria at least the reality appears markedly different. We found that medical doctors are the dominant stakeholders in all the different stages of policy-making in the CCM and that the CCM itself further entrenches this.

 Another consequence was the general concern among participants that medical doctor dominance has led to the relegation of people who represent the communities, such as patient groups, Community Based Organisations and non-medical professionals, referred to in this paper as a repressed group. “*Like the social mobilization and gender issues, (but) we are not focusing and paying more attention to those issues... the medical doctors will continue to have an upper hand” *(Participants 21: Finance expert).

 “*I think because they (non-medical stakeholder) never get-to-get university degrees like the other health professionals. So they do not have that sophistication that MBBS medical doctors will have... it’s probably oppression on the part of the other health professionals playing them down” *(Participant 22: Medical Doctor).

 The themes from this study can be considered in terms of actors (*‘individuals, organizations or even the state and their actions that affect policy’*), content (*‘substance of a particular policy’*) and processes (*‘how policies are initiated, developed or formulated, negotiated, communicated, implemented and evaluated’*).^1^ This approach can be used to power dynamics and has been advocated by others health policy researchers.^36^ However, these relationships are not wholly independent, for instance, actors use power to influence policy processes and content, and the processes themselves influence the decisions that are made.

## Discussion

 This study provides a rich understanding of medical professionals’ dominance and their assertive interactions within policy processes and spaces in the context of the Nigerian health system. Most importantly, this paper highlights how theories from the sociology of professions can be used to explore power during health policy-making. Firstly, the findings provide empirical evidence of medical professionals’ dominance in terms of number and their hold on influential positions^37^ which have previously been poorly understood in this context. In addition, through the content and processes of policy-making, medical professionals are able to maintain these existing professional hierarchies and express influence in the health system. The discursive frequency with which participants noted medical professionals’ influence in the proposal writing implies that medical doctors continue to maintain a professional monopoly.^38-42^ The characterisation of medical professionals as ‘drivers’ of the process by study participants implies that their active participation brings about an unequal influence in agenda setting, thereby extending their scope into non-clinical areas that may require expertise of social scientists, implementers or the patients themselves. These patterns of medical dominance in the health sector have been described by sociologists, such as Freidson and other authors in Western settings^16,43-45^ yet, to the best of our knowledge, they have not been previously explored in sub-Saharan Africa.

 In the literature on professions, Larson argues that professional monopoly is achieved through either monopolisation of the production of knowledge and practice, occupational hierarchy or both.^17^ Occupational hierarchy is a unique feature of a professional bureaucracy which has with it specific accompanying characteristics, such as technical autonomy and professional privileges.^17^ This is common in post-colonial states, which have inherited a ‘professional bureaucrat model’ of medical professionals from colonial regimes.^46^ Our findings illustrate that this ‘professional bureaucrat model’ still exists in the Nigerian health system and influences policy creation. The occupational hierarchy places medical professionals as the head of health units in the public sector, which other health occupations appear to have internalised as the norm. As illustrated by our findings, medical professional influence in public-private partnerships, such as the Global Fund’s health initiative is a result of a diffusion of their public sector influence (and in part their privileged social status) into the Global Fund policy process.

 Shiffman has proposed unravelling how the various forms of epistemic power (in this study, in the form of biomedical discourse dominance) are expressed in policy processes: ‘*Each of these two kinds of assertions—epistemic and normative—invoke both structural and productive power’ *^13^ (p. 297). This has contributed to this ongoing discussion on power in health policy, using the theories from the sociology of professions as another lens in exploring the forms of power highlighted by Shiffman and others. While it is difficult to distinguish between the use of productive and structural power by global health actors, describing the structural form of power discussed by Shiffman and Lee^7,13^ is important because in synergy, these two forms of power can give insight into the dominance of a particular discourse. As highlighted in our study, the Global Fund’s TRP can be seen as a major mechanism through which structural power is exercised by the donor over the recipient, because in situations of negotiations *‘they not only had the money behind them, but also good evidence that theirs was the best for the situation. Hence, a strong argument with robust evidence-based rationale can sway opposing stakeholders’*^47^ (p. 356). The TRP, with its biomedical base, uses the structural power of ‘superior’ evidence to oblige recipient countries to conform to their preferred approach. This reliance on biomedical evidence as the primary source of evidence during the proposal writing process compels CCM members to follow medical guidelines, with some participants describing the process as ‘prescriptive.’ In this way, the TRP at the same time maintains and re-enforces biomedical dominance. This is because though proposals should reflect a country’s priorities, our findings suggest that in-country stakeholders involved in the proposal writing process accept the TRP’s ‘superiority’ in the technical knowledge hierarchy and design their proposals accordingly, thus prioritising information required by the TRP.

 In relation to this study, productive power is seen in the way meetings and deliberative processes are dominated by biomedical language, relegating other forms of reasoning. This finding is similar to *‘unconscious dogmatism’* described by Ooms which denotes how some health actors believe that there is only one (biomedical) way to view health^6^ (p. 643). Of note is that this productive power of the biomedical discourse has been (consciously or unconsciously) exploited by medical professionals in the way in which they use biomedical language at the exclusion of other non-doctor actors from the policy process. Therefore, an argument can be made that this is a form of *‘stealth advocacy’* used by medical professionals to maintain their relevance in policy-making rather than a case of *‘unconscious dogmatism.’* The medical profession in various (other) contexts has been shown to draw upon similar forms of power, in order to gain monopoly and maintain dominance over other health occupations.^10,48-50^ The resulting exclusion of other health workers and patients from certain roles taken up by medical professionals in the policy process has been identified in this study as one of the major reasons for poor implementation outcomes and policy failure.

 The dominant biomedical discourse within the Global Fund’s structure creates technical specifications and institutional procedures that reinforce the opportunities for medical professionals to continue to dominate participation and implementation of the Global Fund grant. The Global Fund has since expanded its secretariat’s country team presence in Nigeria, increasing its foreign oversight function of country activities, while directly negotiating with individual state governments and stakeholders, thereby bypassing the CCM. In essence, the Global Fund is beginning to structurally operate in a way that is akin to other GHIs, such as United States Agency for International Development, Department for International Development and President Emergency Plan for AIDS Relief. This implies that the Global Fund, rather than continuing to (in theory at least) empower health system actors in developing a more robust and inclusive health policy space through the CCM, has opted instead for the short-term benefit of proactively steering the strategy of the country grant towards their own donor targets. With this shift, the Global Fund could risk further alienating non-medical CCM stakeholders who would not have been able to be included in the health policy-making space without the CCM.

 Due to the sensitivity of the research, the researchers may have been unaware of some forms of exclusion, such as not being invited to social events, the use of language that the observer does not understand or even participants moving away from the researcher when having serious conversations.^51^ The snapshot nature of the cross-sectional observations is a limitation, as it is difficult to observe most of the experiences of participants and the evolution of some of these phenomena over time.^52^ However, the interview data were triangulated with observations and policy documents to make the findings more robust. Another limitation is that the study focused only on the Global Fund’s CCM which limited the sample size. The Global Fund, however, is a major donor in Nigeria and the study involved many of the current and previous stakeholders in the CCM. Although the study was conducted pre-COVID-19 (coronavirus disease 2019) times, anecdotal evidence suggests not much is likely to have changed. If any, perhaps the biomedical discourse is becoming even more dominant and powerful as a result of COVID-19 vaccines leading the global health debate.

 Finally, this study has uncovered the influence of medical professionals in the Nigerian health system, stretching beyond simply shaping the implementation of national health policies, to also altering the policy content and process.^53^ Health systems in LMICs are multi-professional and multi-disciplinary, involving various actors that interact with the system in complex ways and sometimes in opposition to each other.^53^ However, as illustrated through this case study, the Nigerian health system remains strongly dominated and guided by biomedical and clinical discourses because the *‘dominant group of actors (in terms of both volume and influence) are those involved in the delivery of health services, primarily medical professionals’ *^54^ (p. 4). Both the productive and structural power of the biomedical discourse as seen in this study are contributory factors to the Nigerian medical professional monopoly and how this monopoly reinforces biomedical discourse in the content and processes of health policy. This link between the sociology of professions and theories of power (seen in Table 1) improves our understanding of power in relation to health policy and how professional monopoly forms a major part of policy formulation in the context of Nigeria. Importantly these unequal distributions of power at the national level do not occur in isolation from the local level where inter-professional conflicts are more evident. For this reason the study of power dynamics among health professionals is imperative in understanding policies and practice in LMICs. We call for further research to explore the diffusion of biomedical power from the global to the national and local levels and vice versa, particularly in post-colonial settings.

## Conclusion

 This study reveals how the Global Fund structure interacts with and influences medical professional powers within the Nigerian health system. In particular, the study illustrates the different forms of power among professional actors involved in the health policy process of the CCM in Nigeria. In conclusion, creating more open inter-disciplinary policy spaces would allow active participation of repressed interests that represent both the patient population and other non-clinical professions. This is only possible through the sensitisation of the non-health sector ^55^ and the removal of structural barriers put in place to protect the jurisdiction of dominant professional monopolies.^56^

## Acknowledgements

 This work is part of Dr. Samuel Lassa’s PhD thesis in the School of Health and Health Related Research, University of Sheffield. We would like to acknowledge the CCM in Nigeria for supporting the research team. Staff of The Department of Community Medicine, University of Jos, Nigeria for being part of the data gathering team and ethics application.

## Ethical issues

 Ethical approval was obtained from the National Health Research Ethics Committee, Nigeria of the Ministry of Health in Nigeria through the University of Jos and the School of Health and Health Related Research (ScHARR) Research Ethics Committee at the University of Sheffield, UK. The CCM in Nigeria also gave approval and consent for their members to be recruited for interviews. Individual written informed consent was obtained from each participant prior to data collection.

 The principal researcher kept detailed daily field notes during interview and observation data collection. The research team used regular research meetings to limit the effect of bias in the data by reviewing interview recordings and field notes during every stage of data collection. Through a conscious reflexive approach, the research team evaluated all the research processes and invited experienced external researchers familiar with the context and topic to impartially examine the data quality and interpretations.

## Competing interests

 Authors declare that they have no competing interests.

## Authors’ contributions

 Conception and design: JO, SL, and JB. Acquisition of data: SL. Analysis and interpretation of data: SL, JO, and JB. Drafting of the manuscript: SL, CB, JB, and MS. Critical revision of the manuscript for important intellectual content: JB, MS, and CB. Supervision: JO, JB, and MS.

## Funding

 Tertiary Education Trust Fund Nigeria PhD scholarship awarded to Dr. Samuel Lassa to carry out this study.

## Endnotes


^[1]^ In this paper we use the following working definition of policy failure, namely that a policy fails even if it is successful in some minimal way, if it does not fundamentally achieve the goals its proponents set out to achieve.
^[2]^ In the Global Fund, the TRP is the technical arm, designed to guide and inform the board during decision making.^57,58^
